# *Circ_0088200* acts as a sponge for miR-127-5p to promote the migration and invasion of rheumatoid arthritis fibroblast-like synoviocytes

**DOI:** 10.1515/rir-2025-0002

**Published:** 2025-04-02

**Authors:** Yujie Cai, Rong Qiu, Qin Huang, Weinan Lai, Yipeng Han, Xiaoxi Lu, Jiayu Qin, Qingqing Ouyang, Min Yang

**Affiliations:** Department of Rheumatology and Immunology, Nanfang Hospital, Southern Medical University, Guangzhou 510510, Guangdong Province, China

**Keywords:** rheumatoid arthritis, circRNA, fibroblast-like synoviocytes, miR-127-5p, matrix metalloproteinase 1

## Abstract

**Background:**

Circular RNAs (circRNAs) play a crucial role in the development of various diseases. However, few studies have investigated the role of circRNAs in rheumatoid arthritis (RA). Herein, we aimed to identified the novel circRNAs involved in the migration and invasion of RA fibroblast-like synoviocytes (RA-FLS).

**Methods:**

The RA-FLS were isolated from the synovial membrane of patients with RA. The CircRNA profile was screened by CircRNA microarray analysis. *Circ_0088200* and miR-127–5p expression levels were detected using quantitative real-time reverse transcription polymerase chain reaction (qRT-PCR). The protein level of matrix metalloproteinase 1 (MMP1) was evaluated by western blotting. Wound healing and Transwell assays were performed to analyze the migration and invasion of RA-FLS. RNA immunoprecipitation (RIP) and dual-luciferase reporter assays were used to validate the interaction between *Circ_0088200* and miR-127–5p. Collagen-induced arthritis (CIA) mouse models were established to evaluate the role of *Circ_0088200* in the development of arthritis *in vivo*.

**Results:**

*Circ_0088200* was highly expressed in RA-FLS compared with osteoarthritis fibroblast-like synoviocytes (OAFLS) and correlated positively with the disease activity score in 28 joints. Inhibition of *Circ_0088200* suppressed the migration and invasion of RA-FLS. Conversely, overexpression of *Circ_0088200* significant promoted the migration and invasion of RA-FLS. Mechanistically, *Circ_0088200* functions as a sponge for miR-127–5p and relieve its repressive effect on *MMP1*, thereby promoting the migration and invasion of RA-FLS. Importantly, intra-articular injection of Adenoassociated virus expressing *Circ_0088200* significantly increased the severity of arthritis in mice with CIA.

**Conclusion:**

*Circ_0088200* promotes the migration and invasion of RA-FLS by sponging miR-127–5p. Thus *Circ_0088200* is a potential therapeutic target for RA.

## Introduction

Rheumatoid arthritis (RA) is the most prevalent chronic inflammatory disease, characterized by chronic synovial inflammation and joint structure destruction.^[1]^ Although the use of biological agents has greatly improved the treatment of RA, some patients still show a poor response to currently available treatment.^[2,3]^ Therefore, it is necessary to investigate the pathogenesis of RA and identify potential therapeutic targets.

RA-affected joints mainly present with synovial inflammation. In these joints, fibroblast-like synoviocytes (FLS) in the syno-vial lining layer play a pivotal role in the pathogenesis of RA.^[4]^ During RA inflammation, activated FLS show biological behavior similar to tumor cells, and their migration and invasion are significantly enhanced.^[5]^ RA-FLS migrate to the surface of bone and cartilage in response to a variety of inflammatory mediators and cytokines, and produce proteolytic enzymes, resulting in erosion of cartilage and bone, and destruction of joints.^[6]^ Moreover, activated RAFLS can also migrate to distant joint inflammation sites and have a certain impact on the destruction of multiple joints.^[7]^ However, the molecular mechanism underlying the migration and invasion of RA-FLS remain largely unknown.

Circular RNAs (circRNAs) are a class of RNA molecule with a closed loop structure formed by covalent bonds, which do not have a 5’ terminal cap and a 3’ poly A tail.^[8]^ They are widely present in eukaryotic cells and show tissue specificity, disease specificity, and timing specificity.^[9]^ Numerous studies have revealed the important role of circRNAs in a variety of diseases, including cancer, osteoarthritis (OA), and autoimmune diseases.^[10, 11, 12]^ For instance, *CircFNDC3B* is upregulated in oral squamous cell carcinoma (OSCC) and is associated with lymph node metastasis; *CircFNDC3B* sequesters miR-181c-5p to induce epithelial–mesenchymal transition, migration, and invasion of OSCC cells.^[13]^ *CircRPN_2_* is down-regulated in hepatocellular carcinoma (HCC) and inhibits the migration and invasion of HCC cells; *CircRPN_2_* inhibits glucose metabolism by acting as a competing endogenous RNA (ceRNA) for miR-183–5p, thereby inhibiting HCC cell migration and invasion.^[14]^ RA-FLS have unique aggressive behaviors similar to cancer cells and play a key role in the pathogenesis and progression of RA. However, few studies have investigated the roles of circRNAs in the migration and invasion of RA-FLS. In our previous study, we used a cir-cRNA microarray to analyze the differential expression profile of circRNAs between RA-FLS and OA-FLS.^[15]^ We identified that circRNA *Circ_0088194* could promote the migration and invasion of RA-FLS. *Circ_0088194* acts as an miR-766–3p sponge to relieve the repressive effect on its target mRNA, *MMP2* (encoding matrix metalloproteinase 2 protein), thus promoting migration and invasion.

In the present study, we continued to investigate the differentially expressed circRNAs between RA-FLS and OA-FLS. We identified that *Circ_0088200* was significantly upregulated in RA-FLS and involved in the migration and invasion of RAFLS. *Circ_0088200* is a potential therapeutic target for RA.

## Materials and Methods

### Patient Samples and Cell Preparation

Nine RA synovial tissues and seven OA synovial tissues were acquired from patients with end-stage symptomatic hip RA or OA at the time of total hip replacement surgery, as performed at the Department of Orthopedic Surgery, Nanfang Hospital, Southern Medical University, Guangzhou, China. Patients with RA or OA were diagnosed according to the 2010 American College of Rheumatology/European League Against Rheumatism (ACR/EULAR) classification criteria or the 1995 American College of Rheumatology (ACR) classification criteria, respectively.^[16,17]^ The Ethics Committee of the Southern Medical University approved the study and its associated protocols (Guangzhou, China, NFEC-20120201). All participants provided written informed consent. The clinical characteristics and laboratory measures of the participants are listed in Supplementary Table 1.

### CircRNA Microarray Analysis

Sample labeling and array hybridization were performed according to the manufacturer’s protocol (Arraystar Inc., Rockville, MD, USA). The microarray analysis was carried out by KangChen Bio-tech, Shanghai, China. More details about CircRNA microarray analysis are provided in Supplementary Methods and Materials.

### Culture of FLS

FLS were isolated from human synovial tissue specimens and cultured in Dulbecco’s modified Eagle’s medium (DMEM) (Thermo Fisher Scientific Inc., Waltham, MA, USA) with 10% added fetal bovine serum (FBS)(Gibco BRL, Grand Island, NY, USA). FLS were used from passage four to six, during which time they were confirmed using vimentin immunofluo-rescent staining as a homogenous population with a purity > 98%.

### Quantitative real-time polymerase chain reaction (qRTPCR) Analysis

Total RNA was obtained from cultured cells using the TRIzol reagent (Takara, Shiga, Japan), according to the manufacturer’s instructions, and reverse transcribed into cDNA. The qRTPCR step was carried out using the cDNA as the template and SYBR Premix DimerEraser (Takara). Supplementary Table 2 shows the details of the primers used for qRT-PCR. The details about qRT-PCR analysis are provided in Supplementary Methods and Materials.

### Electrophoresis of Nucleic Acids

Agarose gel electrophoresis (4%) with Tris acetate ethyl-enediaminetetraacetic acid running buffer (Thermo Fisher Scientific) was used to analyze genomic DNA (gDNA), PCR products, and cDNAs. Electrophoresis was performed at 110 V for 50 min to isolate DNA. A 20 bp DNA marker (Takara) was used and bands were examined under ultraviolet irradiation.

### Fluorescence In Situ Hybridization

Fluorescence *in situ* Hybridization (FISH) analysis of RAFLS used biotin-labeled probes specific to *Circ_0088200* (GenePharma Co. Ltd., Shanghai, China). We used FISH (GenePharma) to detect the signals of these probes according to the manufacturer’s instructions. 4, 6-diamidino-2-phe-nylindole (DAPI) was used to counterstain the nuclei. A Leica TCS SP2 AOBS confocal microscope (Leica Microsystems, Mannheim, Germany) was used to acquire images.

### Western Blotting Analysis

Cultured RA-FLS were lysed in ice-cold radioimmunoprecipi-tation assay buffer (BestBio, Shanghai, China). The proteins in the cell lysates were separated using 10% SDS-PAGE, followed by electroblotting onto a polyvinylidene difluoride membrane (Millipore, Billerica, MA, USA). The signals from the immunoreactive proteins were quantified using Quantity One Software (Bio-Rad, Hercules, CA, USA). The details about western blotting analysis are listed in Supplementary Methods and Materials.

### Adenoviral Vector Construction and in Vitro Transduction

The adenoviral expression vector for *Circ_0088200* over-expression was constructed by Genepharma (Shanghai, China). For overexpression of *Circ_0088200*, the full length *CircRNA_0088200* sequence was inserted into the pHBADVCMV-circ-EF1-ZsGreen vector (to form Adv *Circ_0088200*), with an empty Adv vector being used as the control. The target and primer sequences for the circRNA are listed in Supplementary Table 2. The transfection process was performed using Lipofectamine 3000 (Hanbio Biotechnology, Shanghai, China) according to the manufacturer’s protocol. The expression levels of *Circ_0088200* were determined using qRT-PCR.

### Oligonucleotides and siRNA Transfection

Small interfering RNAs (siRNAs), miRNA mimics, and miRNA inhibitors were obtained from RiboBio (Guangzhou, China). Cells were transfected with 50 nM of *Circ_0088200* siRNAs, *MMP1* siRNAs, miR-127–5p mimics, miR-127–5p inhibitors, or the corresponding controls using RNAiMAX (RiboBio) according to the manufacturer’s instructions. All relevant sequences are listed in Supplementary Table S2.

### Cell Migration and Invasion Assays

For the migration assay, 1 × 10^4^ cells in 200 mL of DMEM (serum free) were seeded into the top chamber of a Transwell insert, and then DMEM with 10% FBS (600 mL) was added to the bottom chamber. The invasion assay started the same, except that in addition to the above, 50 μL of Matrigel was layered onto the top chamber. More details about cell migration and invasion assays are provided in Supplementary Methods and Materials.

### RNA Immunoprecipitation

RNA immunoprecipitation (RIP) experiments were conducted following the instructions of the Magna RIP RNA-Binding Protein Immunoprecipitation Kit (Millipore, Bedford, MA, USA). More details about RNA Immunoprecipitation are available in Supplementary Methods and Materials.

### Microarrays Analysis

Microarray analysis was performed on RA-FLS transduced with the adenoviral vector encoding *Circ_0088200* and RAFLS transduced with adenoviral empty vector. Agilent Human 4x44K Gene Expression Microarrays v2 was performed to analysis the global gene expression profile. The details about microarrays analysis are listed in Supplementary Methods and Materials.

### Dual-luciferase Reporter Assay

A *Circ_0088200* segment and a fragment of the *MMP1* mRNA were synthesized with either mutant (MUT) or wild-type (WT) miR-127–5p seed regions and cloned into the pmirGLO vector (Promega, Madison, WI, USA). RA-FLS (1 × 105) were transfected with either the WT or MUT *Circ_0088200*, miR-127–5p mimics or mimics control, and WT or MUT *MMP1* mRNA. The luciferase activity was assessed using a dualluciferase reporter kit (Promega). The detailed information is provided in Supplementary Methods and Materials.

### Bioinformatic Analysis

Potential miRNA targets were predicted using two publicly available databases: TargetScan (https://www.targetscan.org/vert_80/) and circular RNA interactome (https://circinter-actome.nia.nih.gov). Targets were accepted only when they appeared in both databases.

### Experimental Collagen-Induced Arthritis (CIA) Mouse Model

All animal-related experiments in this study were performed with approval from the Medical Ethics Committee of Nanfang Hospital and carried out in strict compliance with the animal ethics requirements. A model of CIA was induced by intra-dermal immunization with emulsions prepared using Bovine type II collagen (CII) and complete Freund’s adjuvant (CFA, Sigma, Kawasaki, Japan) in a 1: 1 ratio. More details are listed in Supplementary Methods and Materials.

### Articular Injection of Circ_0088200

The Adeno-associated virus vector encoding *Circ_0088200* (AAV-*Circ_0088200*) and Adeno-associated virus empty vector were constructed and packaged by Hanbio Biotechnology. A 10 μL solution containing the experimental virus or control virus was slowly injected into each knee joint on the 28 day. The injection procedure was repeated after 1 week and the mice were sacrificed at 60 days. The details about articular injection of Circ_0088200 are available in Supplementary Methods and Materials.

## Histological Analysis, H&E Staining, and Safranin O-fast Green Staining

The arthritis scores and measurement of paw thickness were carried out 2–3 times per week beginning from day 27. Each section (see below) was assessed by two blinded, independent graders, and the average score was used for statistical analysis. On day 60 after the first immunization, the hind paws were dissected from sacrificed mice and fixed in 4% paraformaldehyde (Reagen Technology Co. Ltd., Beijing, China) for paraffin embedding. Subsequently, each paraffin-embedded sample was sectioned at 5 μm, and the sections were stained with hematoxylin-eosin (H&E) and Safranin O-fast Green solution (Guangshi Reagent Technology Co. Ltd., Guangzhou, China) according to standard methods.

### Immunohistochemical Staining

For immunohistochemistry (IHC), the sections were incubated at 4 °C overnight with specific antibodies (Zeye Technology Co. Ltd, Shanghai, China) and then for 30 min at room temperature with secondary antibodies (Hongmai Biotechnology Co. Ltd., Guangzhou, China). Subsequently, a 3, 3′-Diaminobenzidine (DAB) color development kit (Zeye Technology) solution was dropped onto to each section and incubated for 2 min at room temperature for staining.

### Micro-Computed Tomography (CT) Analysis

After the specimens were processed in 4% paraformalde-hyde, the fixed knee samples were evaluated in a scanning tube with a volex size of 8.8 μm and scanned for 40 min of acquisition time using a SCANCO high-resolution MicroCT instrument (SCANCO Medical AG, Brüttisellen, Switzerland). During scanning, the samples were wrapped in a paper soaked in PBS to avoid dehydration, and the data were analyzed using Data View software (Chauvin Arnoux UK Ltd., Dewsbury, UK).

### Statistical Analysis

All experiments were carried out three times. Data are shown as the mean ± the standard deviation (SD). For comparisons between two groups or among multiple groups, we used Student’s t-test and one-way analysis of variance (ANOVA), respectively. Spearman rank correlation was used to analyze the association of *Circ_0088200* expression with disease activity. All statistical analyses were performed using the SPSS 20.0 software (IBM Corp. Armonk, NY, USA), and graphs were drawn using GraphPad Prism 7.0 (GraphPad Software Inc., La Jolla, CA, USA). *P <* 0.05 was considered statistically significant.

## Results

### Characterization and Expression Analysis of Circ_0088200

**Figure 1:**
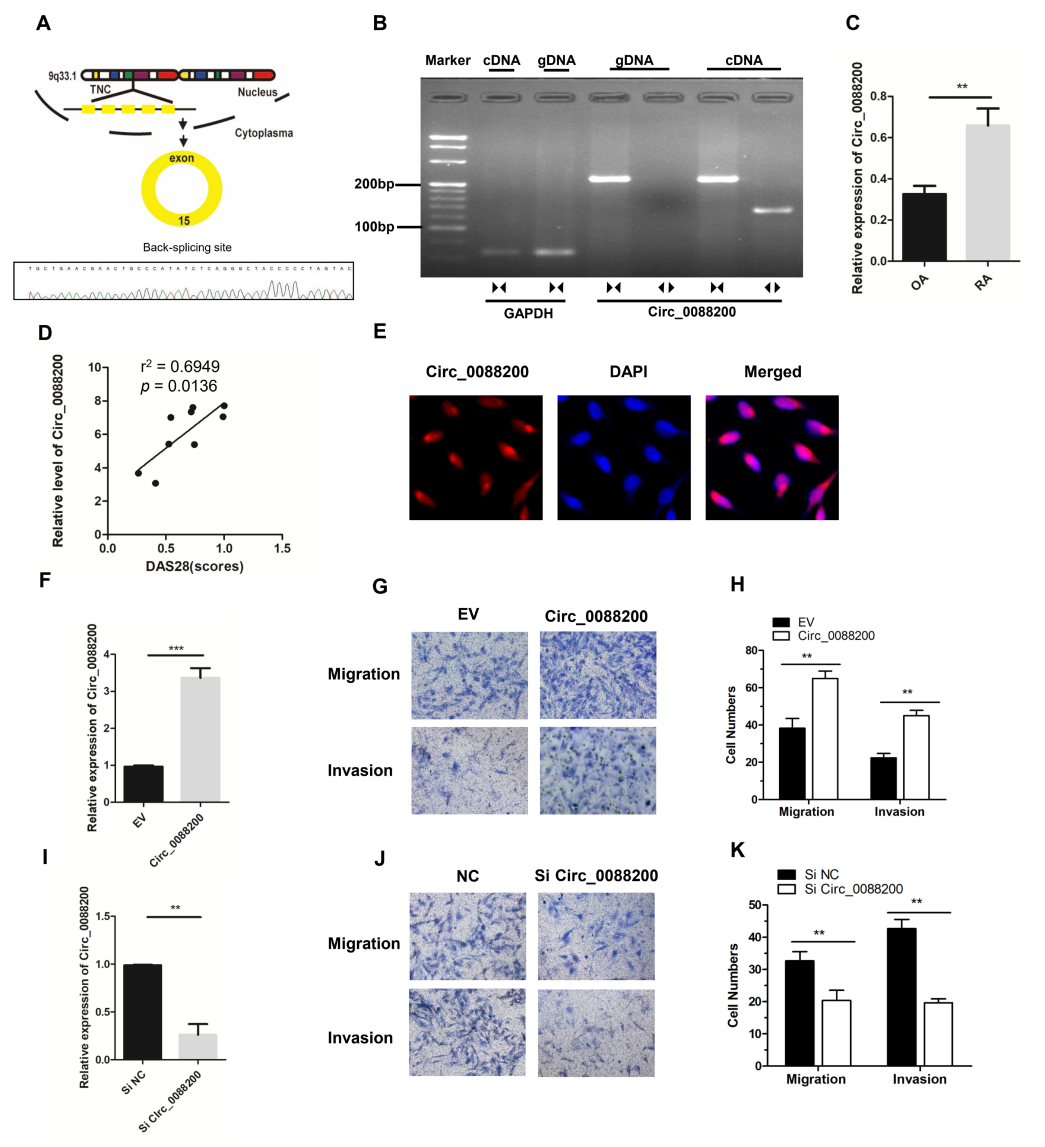
Circ_0088200 promotes the migration and invasion of RA-FLS. (A) Schematic diagram of Circ_0088200 formation via the circularization of exon 15 of TNC (encoding tenascin C). (B) Divergent primers amplified Circ_0088200 from cDNA but not from genomic DNA (gDNA). (C) The expression levels of Circ_0088200 were detected using qRT-PCR. RA-FLS, n = 9; OA-FLS, n = 7. (D) Circ_0088200 expression in RA-FLS correlated positively with the RA disease activity score in 28 joints (DAS28 score). (n = 9; r2= 0.6949, P = 0.0136). (E) The localization of Circ_0088200 in RA-FLS was detected using FISH. Scale bar, 50 μm. (F) RA-FLS were transduced with adenovirus expressing Circ_0088200. The expression of Circ_0088200 was detected using qRT-PCR. (G-H) Migratory and invasive activities of RA-FLS were assessed by Transwell migration and Matrigel invasion assays. (I) The expression of Circ_0088200 was detected using qRT-PCR. RA-FLS were transfected with Circ_0088200 siRNAs. (J-K) Migratory and invasive activities of RA-FLS were assessed by Transwell migration and Matrigel invasion assays. Data are shown as the mean ± SD. NS: not significant. *P < 0.05, **P < 0.01, ***P < 0.001.

In our previous study, we performed circRNA microarray to analyze the differential expression circRNAs between RAFLS and OA-FLS.^[15]^ We identified that twelve circRNAs that were significantly differentially expressed (fold change > 2.5 and *P <* 0.05), including seven upregulated and five downregulated circRNAs in RA-FLS compared with OA-FLS. Among the differentially expressed circRNAs, *Circ_0088200* which was 3.50-fold upregulated in RA-FLS, attracted our attention. The gene encoding *Circ_0088200* is located at chr9: 117819431–117819704 and *Circ_0088200* is formed by the reverse splicing of exon 15 of the *TNC* (tenascin C) gene (Figure 1A). The existence of *Circ_0088200* was confirmed in numerous circRNA databases. According to the circBase database, *Circ_0088200* exists in many tissue and cell types, such as normal human epidermal keratinocytes. Two groups of primers were designed: Divergent primers were designed to amplify *Circ_0088200*, and convergent primers were designed to amplify the *TNC* mRNA. The cDNA and gDNA samples were used as templates for qPCR. Using the divergent primers, we could amplify *Circ_0088200* using the cDNA as a template, but not using the gDNA. Sanger sequencing of the PCR products amplified using the divergent primers further confirmed the back-splice junction of *Circ_0088200* (Figure 1B). These results indicated that the *Circ_0088200* could be specifically amplified by qRT-PCR and confirmed that *Circ_0088200* exists in RA-FLS.

Next, we expanded the sample size to validate the expression of *Circ_008820*0 in nine RA-FLS and seven OA-FLS samples using qRT-PCR. Compared with that in OAFLS, the expression level of *Circ_0088200* was significantly upregulated in RAFLS (Figure 1C). We found that the *Circ_0088200* expression levels correlated positively with the disease activity score in 28 joints (DAS28)(*r*^2^ = 0.6949, *P* = 0.0136) (Figure 1D). Moreover, a FISH assay revealed that *Circ_0088200* was localized mainly in the cytoplasm of RA-FLS (Figure 1E). Collectively, these results indicated that *Circ_0088200* was significantly upregulated in RA-FLS and correlated with the DAS28.

### Circ_0088200 Promotes Migration and Invasion of RAFLS

To investigate the effect of *Circ_0088200* on migration and invasion of RA-FLS, an adenoviral vector encoding *Circ_0088200* was constructed and siRNAs targeting the junction sites of *Circ_0088200* were designed. We found that the migration and invasion abilities of RA-FLS were significantly increased after transduction with the adenoviral vector encoding *Circ_0088200* (Figure 1F-H). Conversely, the migration and invasion abilities of RA-FLS were decreased after transfection with *Circ_0088200* siRNA (Figure 1I-K). These data demonstrated that *Circ_0088200* promotes the migration and invasion of RA-FLS.

### Circ_0088200 Induces MMP1 Expression in RA-FLS

**Figure 2:**
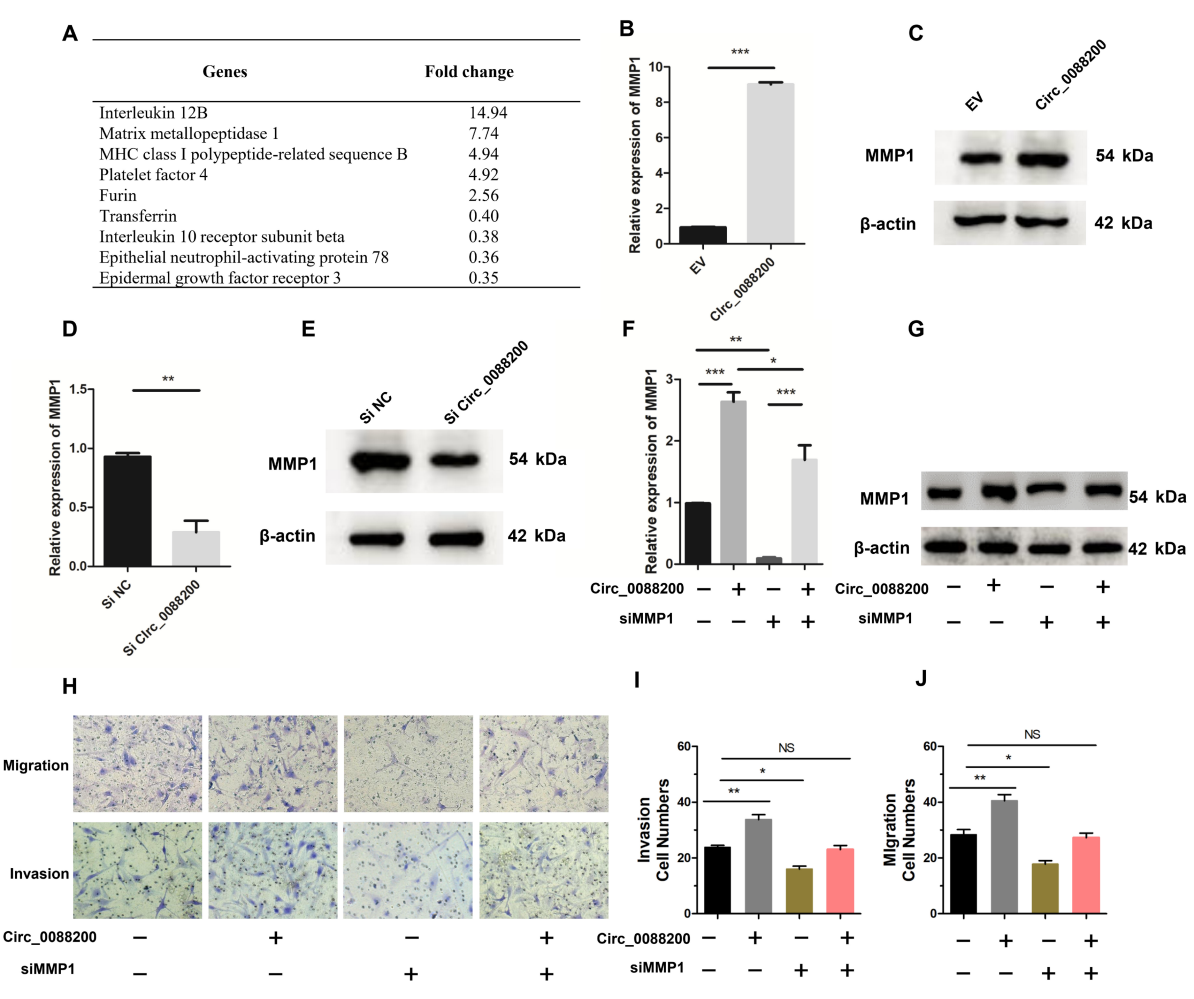
Circ_0088200 induces MMP1 expression in RA-FLS. (A) Microarrays analysis of dysregulated genes in RA-FLS expressing Circ_0088200 and control RA-FLS. (B-C) MMP1 mRNA and protein levels were detected using qRT-PCR and western blotting. RAFLS were transfected with adenovirus expressing Circ_0088200. (D-E) MMP1 mRNA and protein levels were detected using qRT-PCR and western blotting. RA-FLS were transfected with Circ_0088200 siRNA. (F-G) MMP1 mRNA and protein levels were detected using qRT-PCR and western blotting. RA-FLS were cotransfected with the adenovirus expressing Circ_0088200 or/and MMP1 siRNA. (H-J) Migratory and invasive activities of RA-FLS were assessed using Transwell migration and Matrigel invasion assays. RA-FLS were cotransfected with the adenovirus expressing Circ_0088200 or/and MMP1 siRNA. Data are shown as the mean ± SD. NS: not significant. *P < 0.05, **P < 0.01, ***P < 0.001.

To investigate the mechanism by which *Circ_0088200* promotes RA-FLS migration and invasion, we performed microarray analysis to identify the differentially expressed genes in RA-FLS after transduction with the adenoviral vector encoding *Circ_0088200*. We found that five genes were up-regulated and four genes were downregulated (fold change > 2, *P* < 0.05) in RA-FLS transduced with the adenoviral vector encoding *Circ_0088200* (Figure 2A). Among the upregulated genes, *MMP1* aroused our attention. Previous studies have indicated that *MMP1* can promote the migration and invasion of RA-FLS.^[18,19]^ Therefore, we speculated that *Circ_0088200* might promote RA-FLS migration and invasion by increasing the level of *MMP1*. Consistently, we observed that over-expression of *Circ_0088200* increased the expression of *MMP1* at both the mRNA and protein levels (Figure 2B, C). Conversely, inhibition of *Circ_0088200* reduced the mRNA and protein levels of *MMP1* (Figure 2D, E). These results indicated that *Circ_0088200* can promote *MMP1* expression in RA-FLS.

Next, we cotransfected adenoviral vector encoding *Circ_0088200* and the *MMP1* siRNA into RA-FLS, and then assessed the *MMP1* mRNA and protein expression levels, as well as the migration and invasion abilities of RAFLS. Consistently, the upregulation of *MMP1* induced by *Circ_0088200* overexpression was abrogated by transfection with the *MMP1* siRNA (Figure 2F, G). We found that silencing of *MMP1* significant inhibited the migration and invasion of RA-FLS. Importantly, silencing of *MMP1* could block the *Circ_0088200* overexpression-induced migration and invasion in RA-FLS (Figure 2H-J). Taken together, these results demonstrated that *Circ_0088200* promotes the migration and invasion of RA-FLS dependent on *MMP1*.

### Circ_0088200 Sponges miR-127–5p in RA-FLS

**Figure 3:**
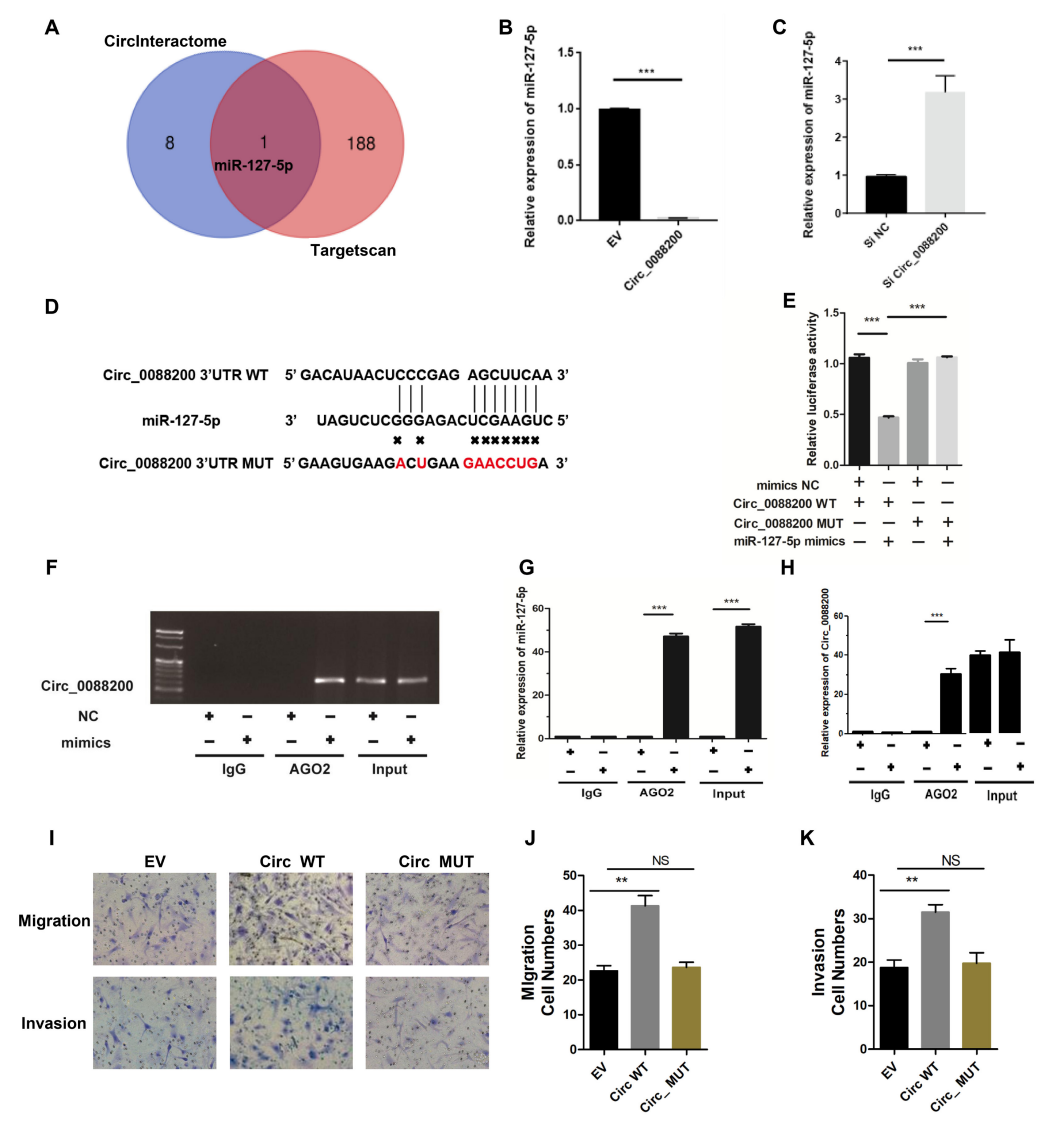
Circ_0088200 acts as sponge of miR-127-5p in RA-FLS. (A) Schematic illustration showing the identification of miR-127-5p, predicted from two available databases (circinteractome and Targetscan). (B-C) qRT-PCR analysis of the expression of miR-127-5p after transfection with adenovirus expressing Circ_0088200 or Circ_0088200 siRNA. (D) Wild-type (WT) or mutant (MUT) miR127-5p target sequences of Circ_0088200. (E) The relative luciferase activities were detected in RA-FLS after co-transfection with luciferase reporter plasmids containing WT or MUT Circ_0088200 sequences and miR-127-5p mimics. (F) RA-FLS were transfected with the miR-127-5p mimics or the negative control. Anti-argonaute 2 (AGO2) RNA immunoprecipitation (RIP) was used to investigate AGO2 binding to Circ_0088200 and miR-127-5p; IgG was used as a negative control. (G-H) The expression of Circ_0088200 and miR-127-5p were detected by qRT-PCR. (I-K) Migratory and invasive activities of RA-FLS were assessed using Transwell migration and Matrigel invasion assays. RA-FLS were transfected with the adenovirus expressing Circ_0088200 or the adenovirus expressing Circ_0088200 with mutated miR-127-5p binding sites. Data are shown as the mean ± SD. NS: not significant. *P < 0.05, **P < 0.01, *** P < 0.001.

CircRNAs located in the cytoplasm might competitively bind to miRNAs and subsequently regulate the expression of their target genes by acting as miRNA sponges.^[20]^ *Circ_0088200* is mostly localized in the cytoplasm of RA-FLS. Therefore, we hypothesized that *Circ_0088200* functions as a miRNA sponge in RA-FLS. Two publicly available databases (TargetScan and circular RNA interactome) indicated that only miR-127–5p could bind to both *Circ_0088200* and the 3′-untranslated region (UTR) of *MMP1* mRNA (Figure 3A). Consistently, overexpression of *Circ_0088200* decreased the level of miR-127–5p; while inhibition of *Circ_0088200* increased the level of miR-1275p in RA-FLS (Figure 3B, C).

To confirm the binding between miR-127–5p and *Circ_0088200*, we performed dual-luciferase assays. The results showed that the miR-127–5p mimics decreased the luciferase activity of the wild-type *Circ_0088200* vector, but not that of the mutant *Circ_0088200* vector (Figure 3D, E). Furthermore, we performed an RIP experiment using anti-argonaute 2 (AGO_2_) antibodies to explore whether *Circ_008820* could serve as a binding platform for AGO_2_ and miR-127–5p. The results showed that *Circ_0088200* and miR127–5p were enriched in the AGO_2_ immunoprecipitates compared with those in the IgG immunoprecipitates (Figure 3F-H). Importantly, overexpression of *Circ_0088200* with mutated miR-127–5p binding sites had no significant effect on the migration and invasion of RA-FLS (Figure 3I-K). Collectively, our findings suggested that *Circ_0088200* acts as a sponge for miR-127–5p.

### miR-127–5p Inhibits Migration and Invasion by Targeting MMP1

**Figure 4:**
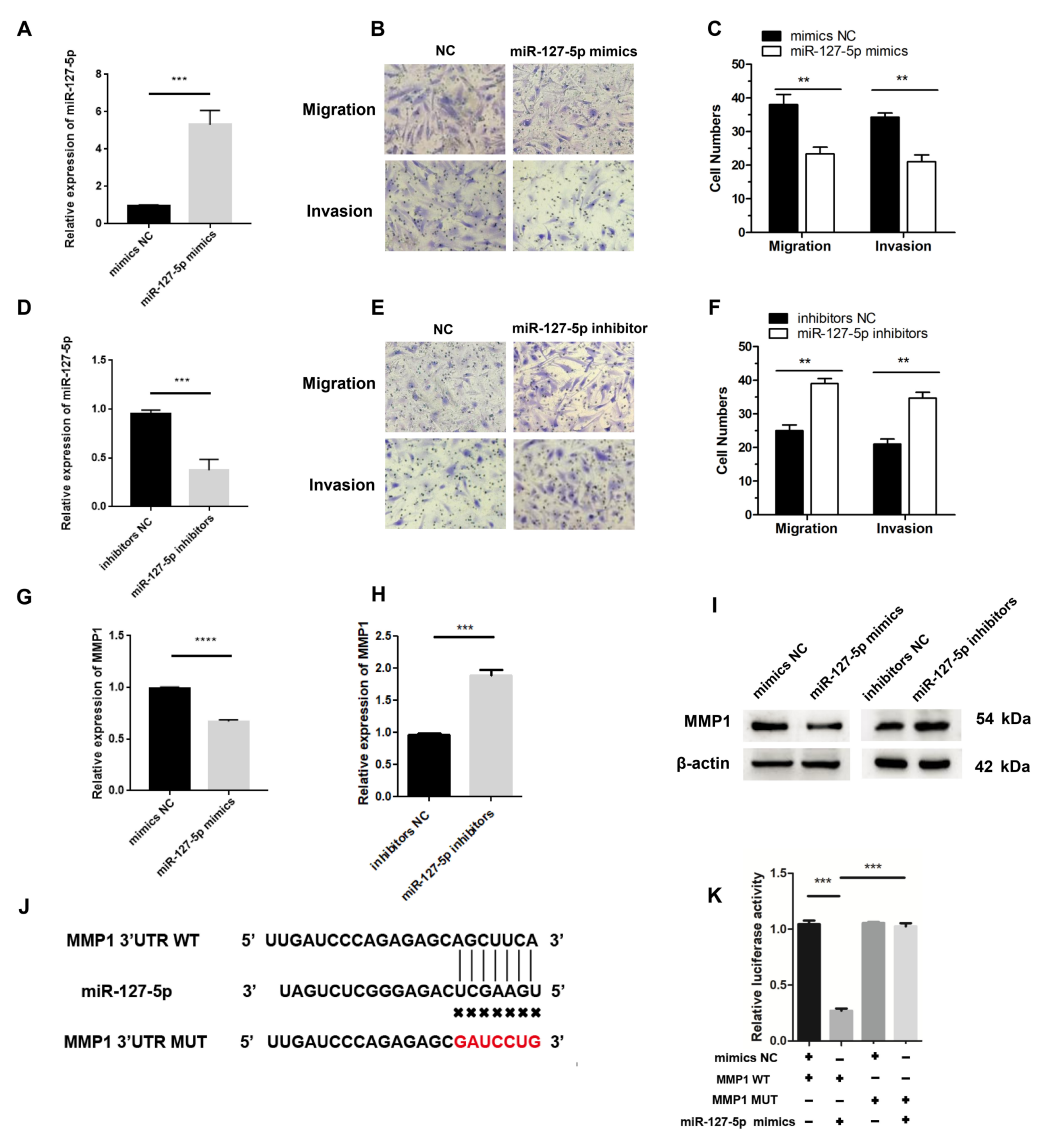
miR-127-5p inhibits RA-FLS migration and invasion. (A) RA-FLS were transfected with the miR-127-5p mimics or the negative control (NC). The expression level of miR-127-5p was determined using qRT-PCR. (B-C) Migratory and invasive activities of RA-FLS were assessed using Transwell migration and Matrigel invasion assays. RA-FLS were transfected with miR-127-5p mimics. (D) The expression level of miR-127-5p was analyzed by qRT-PCR. RA-FLS were transfected with miR-127-5p inhibitors. (E-F) Migratory and invasive activities of RA-FLS were assessed using Transwell migration and Matrigel invasion assays. RA-FLS were transfected with miR-127-5p inhibitors. (G-H) MMP1 mRNA expression was detected using qRT-PCR. RA-FLS were transfected with miR-127-5p inhibitors or miR-127-5p mimics. (I) Western blotting analysis of MMP1 levels. RA-FLS were transfected with miR-127-5p inhibitors or miR-127-5p mimics. (J) Wild-type (WT) or mutant (MUT) miR-127-5p target sequences in the MMP1′ untranslated region (UTR). (K) The relative luciferase activities were detected in RA-FLS co-transfected with luciferase reporter plasmids containing WT or MUT MMP1 sequences and miR-127-5p mimics. Data are shown as the mean ± SD. NS: not significant. * P < 0.05, ** P < 0.01, ***P < 0.001.

To investigate whether miR-127–5p is involved in RA-FLS migration and invasion, RA-FLS were transfected with miR-127–5p mimics or inhibitors. Overexpression of miR-127–5p reduced RA-FLS migration and invasion (Figure 4A-C). Conversely, transfection with the miR-766–3p inhibitors increased the migration and invasion of RA-FLS (Figure 4D-F). Moreover, transfection of miR-127–5p mimics reduced the level of *MMP1*, whereas transfection of miR-766–3p inhibitors increased the level of *MMP1* (Figure 4G-I). These results indicate that miR-127–5p inhibits RA-FLS migration and invasion as well as downregulates the expression of *MMP1*.

**Figure 5:**
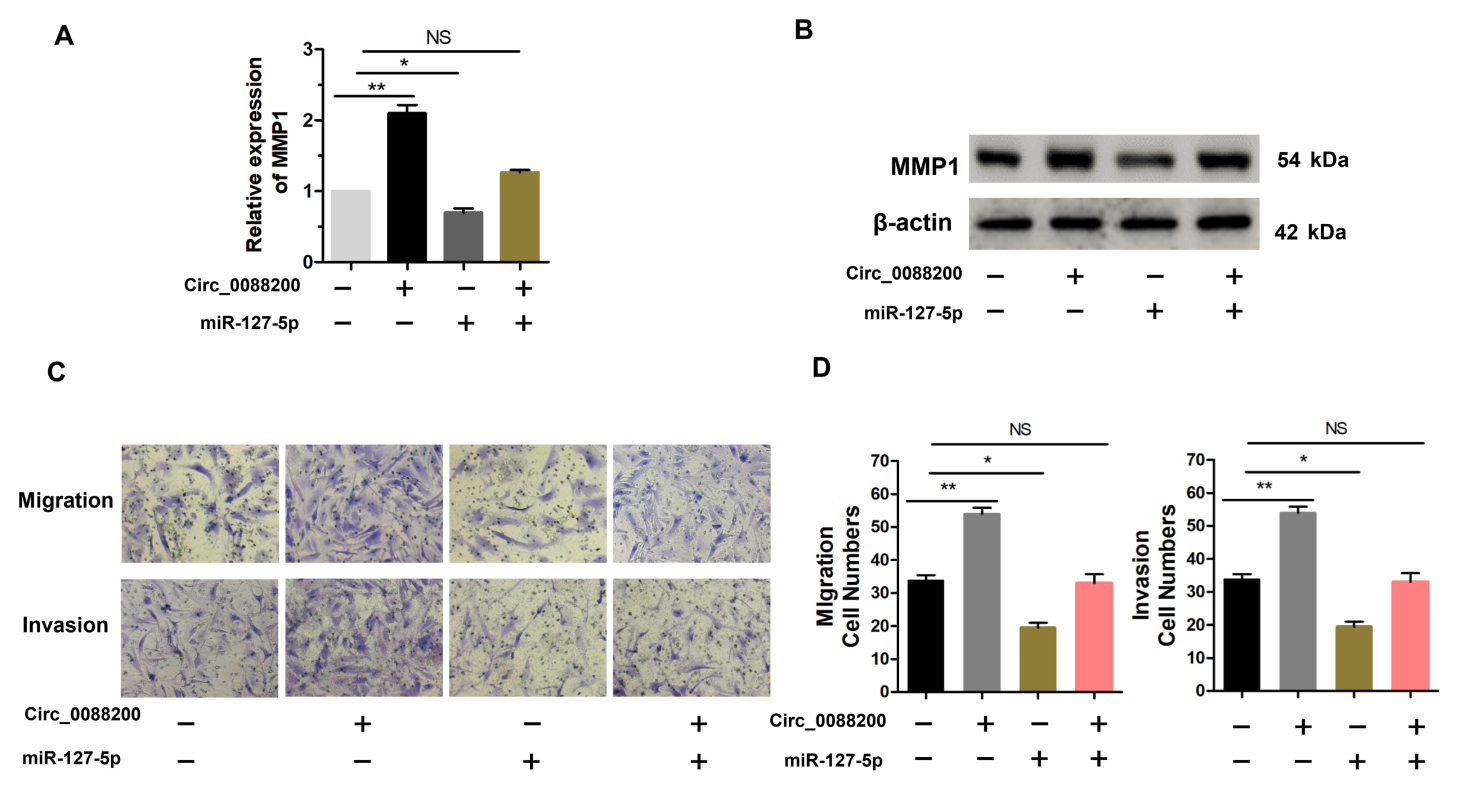
Circ_0088200 promotes RA-FLS migration and invasion via the miR-127-5p/MMP1 axis. (A) The expression of MMP1 mRNA was detected using qRT-PCR in RAFLS cotransfected with miR-127-5p mimics and adenovirus expressing Circ_0088200. (B) Western blotting analysis of MMP1 levels in RA-FLS after cotransfection with miR127-5p mimics and adenovirus expressing Circ_0088200. (C-D) The migratory and invasive abilities of RA-FLS were assessed using Transwell migration and Matrigel invasion assays. RA-FLS were co-transfected with miR-127-5p mimics and adenovirus expressing Circ_0088200. Data are shown as the mean ± SD. NS: not significant. *P < 0.05, **P < 0.01, ***P < 0.001.

Two publicly available databases (TargetScan and circular RNA interactome) predicted *MMP1* is a target of miR-127–5p. To further determine whether miR-127–5p can targeting *MMP1*, we constructed luciferase reporter plasmids containing the WT 3-UTR of the *MMP1* mRNA and a version of the *MMP1* 3UTR in which the miR-127–5p binding sites were mutated, followed by dual-luciferase assays (Figure 4J). The results showed that the miR-127–5p mimics decreased the luciferase activity from the WT construct. However, the mimics had no effect on the luciferase activity from the MUT construct (Figure 5K). These results confirmed that *MMP1* is a target of miR-127–5p. Taken together, our findings implied that miR-127–5p inhibits RA-FLS migration and invasion by targeting *MMP1*.

### Circ_0088200 Promotes RA-FLS Migration and Invasion Via the MiR1275p/MMP1 Axis

Our results indicated that *Circ_0088200* promotes RA-FLS migration and invasion by sponging miR-127–5p, and miR-127–5p promoted RA-FLS migration and invasion by targeting *MMP1*. Therefore, we hypothesized that *Circ_0088200* sponging of miR127–5p might rescue the inhibition of *MMP1* by miR-127–5p, thereby promoting the migration and invasion of RA-FLS. To test this hypothesis, we co-transfected the adenoviral vector encoding *Circ_0088200* and miR-127–5p mimics into RA-FLS. We found that the miR-127–5p mimics blocked the upregulation of *MMP1* induced by *Circ_0088200* overexpression (Figure 5A, B). Consistently, transfection of the adenoviral vector encoding *Circ_0088200* promoted RAFLS migration and invasion, but transfection of miR-127–5p mimics could reverse the migration and invasion induced by *Circ_0088200* overexpression (Figure 5C, D). These results confirmed that *Circ_0088200* promotes RA-FLS migration and invasion *via* the miR-127–5p/*MMP1* axis.

### Circ_0088200 Aggravates the Severity of Arthritis in a Model of RA

**Figure 6:**
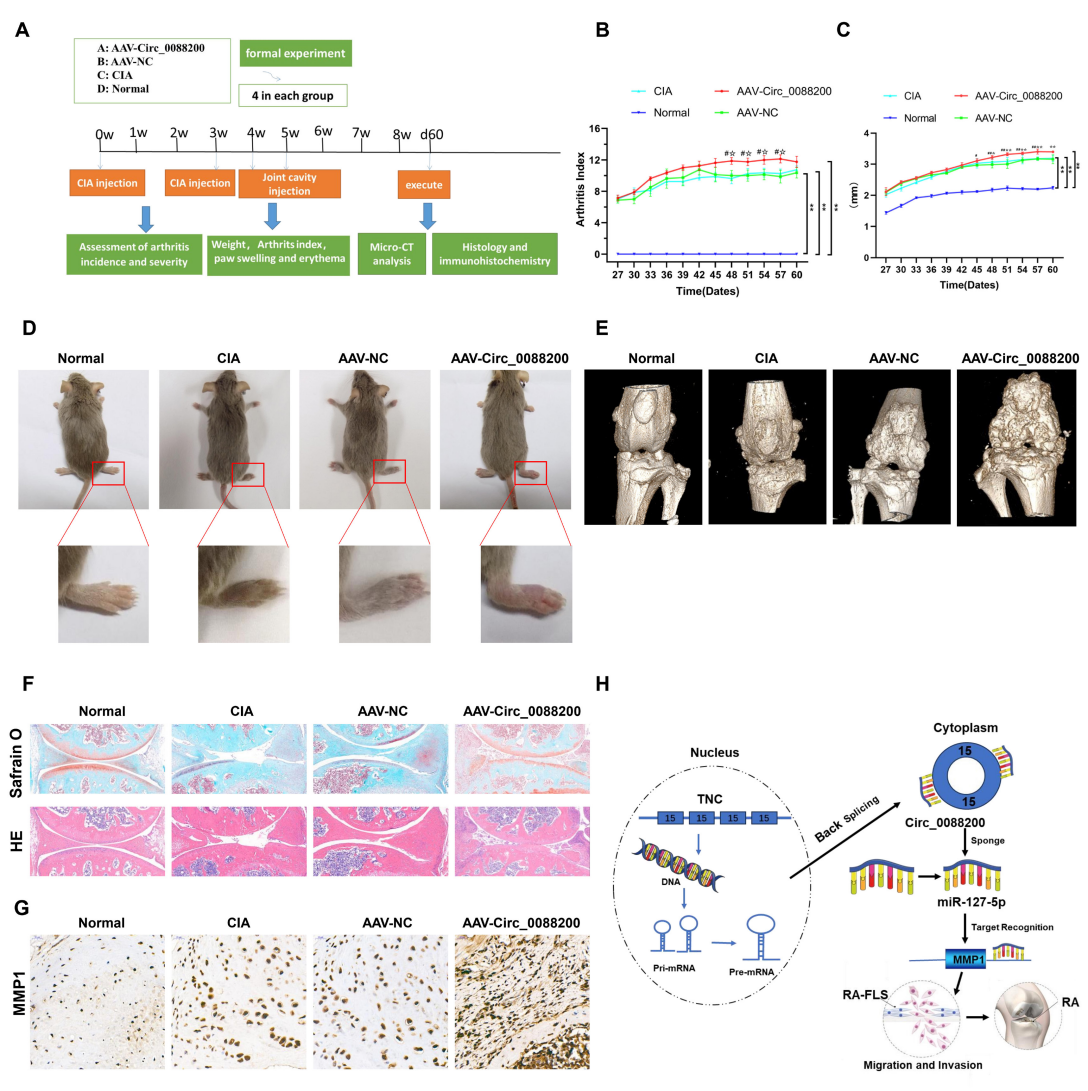
Circ_0088200 aggravates the severity of arthritis in a model of RA. (A) The flowchart for constructing the CIA mouse model (four mice per group). (B) Intra-articular injection of adeno-associated virus vector encoding Circ_0088200 (AAV-Circ_0088200) increased the arthritis scores compared with those in the Adeno-associated virus empty vector (AAV-NC) group and CIA group. (C) Intra-articular injection of adeno-associated virus vector encoding Circ_0088200 increased paw thickness compared with that in the AAV-NC group and CIA group. (D) Representative images of the CIA mouse model. (E) Three-dimensional reconstruction of a CIA mouse knee joint in MicroCT. (F) Representative H& E staining and Safranin O-fast green staining of knee joint sections showing increased cartilage erosions in the AAV-Circ_0088200 group. Scale bar=100 μm. (G) Representative immunohistochemistry sections showing that intra-articular injection of adeno-associated virus vector encoding Circ_0088200 upregulated the expression of MMP1 in the CIA mouse model. Scale bar= 50 μm. (H) Schematic showing that Circ_0088200 promoted the migration and invasion of RA-FLS by sponging miR-127-5p. Data are shown as the mean ± SD. NS: not significant. *P < 0.05, **P < 0.01, ***P < 0.001.

Finally, we established an RA animal model (mice with CIA), to determine the role of *Circ_0088200* in the development of arthritis *in vivo*. The Adeno-associated virus vector encoding *Circ_0088200* (AAV-*Circ_0088200*) or Adeno-associated virus empty vector (AAV-NC) were administered intraarticularly to the CIA mice (Figure 6A). The results showed that the arthritis index (AI) score in the AAV-*Circ_0088200* group was significantly increased compared with that in the AAV-NC group (Figure 6B). Furthermore, we observed that the paw thickness in the AAV-*Circ_0088200* group was significantly increased compared with that in the AAV-NC group (Figure 6C). The AAV-*Circ_0088200* group showed more severe joint redness and swelling (Figure 6D). Moreover, the three-dimensional reconstruction of the micro-CT images demonstrated that intra-articular injection of AAV-*Circ_0088200* promoted cartilage erosion and increased the number of osteophytes (Figure 6E). Additionally, H& E and Safranin O-fast green staining showed that the synovial inflammation and damage to cartilage surfaces in the CIA mice were aggravated after the injection of AAV-*Circ_0088200* (Figure 6F). Importantly, intra-articular injection of AAV-*Circ_0088200* upregulated the expression of *MMP1* in knee joint synovial tissues of mice with CIA (Figure 6G). Taken together, these results indicated that *Circ_0088200* could aggravate the destruction of bone and cartilage tissue in CIA mice models.

## Discussion

CircRNAs are an important part of the human genome, being involved in multiple biological processes.^[10]^ In this study, we identified a novel circRNA, *Circ_0088200*, which was upregu-lated in RA-FLS and can promote the migration and invasion of RA-FLS. Mechanistically, *Circ_0088200* acts as a sponge for miR-127–5p to relieve the repression of *MMP1*, thereby promoting migration and invasion. We demonstrated that the *Circ_00882*00/miR-127–5p/*MMP1* axis plays a key role in the progression of RA (Figure 6H). *Circ_0088200* represent a potential therapeutic target for RA.

CircRNAs are specifically expressed in tissues or cells, have unique molecular structures, are associated with multiple biological processes, and are considered as diagnostic markers and therapeutic targets.^[8]^ Recently, several cir-cRNAs have been shown to be associated with autoimmune diseases, including systemic lupus erythematosus, multiple sclerosis, and RA.^[21,22]^ Studies have focused on dysregulat-ed expression of circRNAs in peripheral blood mononuclear cells in RA.^[23,24]^ However, the roles of most circRNAs in RA development remain unclear. Herein, we demonstrated that *Circ_0088200* promotes the migration and invasion of RAFLS. The *Circ_0088200* expression levels correlated positively with the disease activity score in 28 joints. These results suggested that *Circ_0088200* is a potential prognostic marker for RA.

To better understand RA, several animal models of arthritis have been used.^[25]^ The most common of these is the CIA mouse model, which shows similar clinical, histopathologi-cal, and immunological features to those of human RA.^[26]^ Therefore, we selected the CIA mouse model to investigate the role of *Circ_0088200* in the development of arthritis *in vivo*. Consistent with the results *in vitro*, intra-articular injection of adenoviral expressing *Circ_0088200* aggravated synovial inflammation, joint swelling, and increased bone destruction in CIA mice. These results indicated that *Circ_0088200* plays a pathogenic role in RA progression.

RA-FLS migration and invasion results in erosion of cartilage and bone, and joint destruction.^[27]^ Moreover, activated RA-FLS also can migrate to distant joint inflammation sites and contribute to the destruction of multiple joints.^[28]^ In this study, we used a gene microarray to analyze the potential genes regulated by *Circ_0088200*. We found that overexpression of *Circ_0088200* result in widespread alteration in gene expression and *Circ_0088200* can upregulated the expression of *MMP1*. *MMP1* is expressed robustly by many cell types and degrades all three stromal collagens, making it the foremost player in collagen degradation in many diseases, including cancer and RA.^[29,30]^ Consistently, silencing of *MMP1* significantly attenuated the migration and invasion induce by *Circ_0088200*. Moreover, intra-articular injection of adeno-associated virus expressing *Circ_0088200* in CIA mice significantly increased *MMP1* expression levels in sy-novial tissue. These results indicated that *Circ_0088200* promotes the invasion and migration of RA-FLS a least partially dependent on *MMP1*. However, becasue of the complex and multiple regulation mechanisms of circRNAs, whether *Circ_0088200* can promote the migration and invasion of RA-FLS independent of *MMP1* requires further investigation.

CircRNAs can exert their biological functions by acting as miRNA sponges and regulating binding proteins; however, a few circRNAs also encode proteins.^[8,31]^ CircRNA molecules, which consist of exons and are mainly distributed in the cytoplasm, often exert their functions by acting as miRNA sponges.^[32]^ For example, *CircRNA_000203* is mainly localized in cytoplasm and aggravates cardiac hypertrophy by suppressing miR-26b-5p and miR-140–3p.^[33]^ *CircPTN* sponges miR-145–5p/miR-330–5p to promote the proliferation and stemness in glioma.^[34]^ In the present study, bioinformatic analyses using circinteractome and TargetScan databases showed that miR-127–5p has target sites in both *Circ_0088200* and *MMP1*. We performed luciferase reporter assays and RIP assays to confirm the interaction between *Circ_0088200* and miR-127–5p, as well as between miR-127–5p and *MMP1*. Moreover, miR-127–5p could block the upregulation of *MMP1*, and the migration and invasion induced by *Circ_0088200* overexpression. Thus, we demonstrated that *Circ_0088200* acts as a sponge toward miR-127–5p, which upregulates *MMP1* expression at the post-transcription level, leading to the promotion of RAFLS migration and invasion. To the best of our knowledge, this is the first report that *Circ_0088200* acts as a sponge for miR-127–5p to promote the migration and invasion of RA-FLS. The *Circ_0088200*/miR127–5p/*MMP1* axis plays an important role in the progression of RA. This study improves our understanding of the molecular mechanism of the migration and invasion of RA-FLS.

## Conclusions

We identified that *Circ_0088200* promotes the migration and invasion of RA-FLS. Mechanistically, *Circ_0088200* acts as a sponge of miR-127–5p to relieve the repression of *MMP1* expression, thereby promoting the migration and invasion. We demonstrated that the *Circ_0088200*/miR-127–5p*/MMP1* axis plays a key role in the progression of RA. *Circ_0088200* represents a potential therapeutic target for RA.

## Supplementary Material

Supplementary Material Details
