# Supplementary material for: Circ_0088200 acts as a sponge for miR-127-5p to promote the migration and invasion of rheumatoid arthritis fibroblast-like synoviocytes
Source: Rheumatol Immunol Res. 2025 Apr 2;6(1):7–20. doi: 10.1515/rir-2025-0002 (PMC11966198; doi:10.1515/rir-2025-0002)
Supplement: Supplementary file 1 — Supplementary Material Details [file rir-2025-0002_sm.pdf]

Supplementary Materials and Methods

CircRNA microarray analysis

Sample labeling and array hybridization were performed according to the manufacturer's protocol (Arraystar Inc., Rockville, MD, USA). Briefly, total RNA was digested using RNase R (Epicentre Inc., Madison, WI, USA) to remove linear RNAs and enrich circRNAs. The enriched circRNAs were then amplified and transcribed into fluorescent circRNAs using a random-priming method (Arraystar Super RNA Labeling Kit; Arraystar Inc.) according to the manufacturer’s instructions. Labeled circRNAs were hybridized to the Arraystar Human circRNA Microarray V2.0 (8 × 15K, Arraystar Inc.). After washing the slides, the arrays were scanned using an Agilent G2505C Scanner (Agilent Technologies, Santa Clara, CA, USA). Agilent Feature Extraction software (version 11.0.1.1) was used to analyze the acquired array images. Specific circRNA primers were then used for quantitative real-time reverse transcription polymerase chain reaction (qRT-PCR) analysis, and subsequent data processing was performed using the R software (R version 3.1.2). The microarray analysis was carried out by KangChen Bio-tech, Shanghai, China.

qRT-PCR analysis

Total RNA was obtained from cultured cells using the TRIzol reagent (Takara, Shiga, Japan), according to the manufacturer’s instructions, and reverse transcribed into cDNA. The quantitative real-time PCR (qPCR) step was carried out using the cDNA as the template and SYBR Premix DimerEraser (Takara) with the ABI7500 system (Applied Biosystems, Foster City, CA, USA). Relative expression was calculated using the relative quantification (2-△△Ct) method. *GAPDH* (encoding glyceraldehyde-3-phosphate dehydrogenase) was used as an internal control for circRNAs and mRNAs, and U6 was employed as an endogenous

control for the microRNAs (miRNAs). Supplementary Table 2 shows the details of the primers used for qRT-PCR.

Western blotting analysis

Cultured RA-FLS were lysed in ice-cold radioimmunoprecipitation assay buffer (BestBio, Shanghai, China) containing phosphatase inhibitors and a protease inhibitor cocktail (Sigma-Aldrich, St. Louis, MO, USA). The proteins in the cell lysates were separated using 10% SDS-PAGE, followed by electroblotting onto a polyvinylidene difluoride membrane (Millipore, Billerica, MA, USA). Incubation for 1 hour at room temperature in Tris-buffered saline with Tween-20 and 5% skim milk was used to block non-specific binding to the membrane. The membrane was then probed using primary antibodies recognizing rabbit *MMP1* (1:1000; Abcam, Cambridge, UK) and GAPDH (1:10,000; Bioworld, Bloomington, MN, USA) overnight at 4 °C. Next day, the blot was incubated with horseradish peroxidase-conjugated secondary antibodies (1:10,000; Fdbio science, Hangzhou, China). The signals from the immunoreactive proteins were quantified using Quantity One Software (Bio-Rad, Hercules, CA, USA).

Cell migration and invasion assays

For the migration assay, 1×10^4 cells in 200 ml of DMEM (serum free) were seeded into the top chamber of a Transwell insert, and then DMEM with 10% FBS (600 ml) was added to the bottom chamber. The invasion assay started the same, except that in addition to the above, 50 µl of Matrigel (BD Biosciences, Franklin Lakes, NJ, USA) was layered onto the top chamber. The chambers were then incubated for 48 hours. Thereafter, a cotton swab was used to remove the cells remaining on the surface of the upper membrane surface. Crystal violet was used to stain the cells that had crossed the membrane. Under a microscope, cells in six random fields were counted. These assays were carried out three times independently.

RNA Immunoprecipitation

RNA Immunoprecipitation (RIP) experiments were conducted following the instructions of the Magna RIP RNA-Binding Protein Immunoprecipitation Kit (Millipore, Bedford, MA, USA). Briefly, miR-127-5p mimics or the negative control were transfected into RA-FLS. After 48 hours, the RA-FLS were lysed using Radioimmunoprecipitation assay (RIPA) buffer (Cell Signaling Technology, Danvers, MA, USA). Magnetic beads (Invitrogen, Waltham, MA, USA) were pre-incubated with anti-argonaute 2 (AGO2) antibodies or anti-rabbit IgG (Cell Signaling Technology) for 30 min. The lysates were then immunoprecipitated using the beads, with rotation overnight at 4 °C. Next day, the RNA was purified from the RNA–protein complexes bound to the beads, and then the levels of *Circ_0088200* and miR-127-5p were determined using qRT-PCR, followed by agarose gel electrophoresis.

Microarrays analysis

Microarray analysis was performed on RA-FLS transduced with the adenoviral vector encoding *Circ_0088200* and RA-FLS transduced with adenoviral empty vector. Agilent Human 4x44K Gene Expression Microarrays v2 was performed to analysis the global gene expression profile. Total RNA was extracted from RA-FLS using the TRIzol Reagent (Invitrogen). RNA quantity and quality were measured using a NanoDrop ND-1000 instrument (Nanodrop Technologies. Wilmington, DE, USA). Sample labeling and array hybridization were performed according to the Agilent One-Color Microarray-Based Gene Expression Analysis protocol (Agilent Technology). Agilent Feature Extraction software (version 11.0.1.1) was used to analyze the acquired array images. Quantile normalization and subsequent data processing were performed with using the GeneSpring GX v12.1 software package (Agilent Technologies). Differentially expressed genes with statistical significance

between the two groups were identified through Volcano Plot filtering. Differentially expressed genes between the two samples were also identified through Fold Change filtering.

Dual-luciferase reporter assay

A *Circ_0088200* segment and a fragment of the *MMP1* mRNA were synthesized with either mutant (MUT) or wild-type (WT) miR-127-5p seed regions and cloned into the pmirGLO vector (Promega, Madison, WI, USA). To generate mutant *Circ_0088200* sequences, 15 nucleotides in the seed region were changed (WT seed sequence: 5′-CATAACTCCCGAGAGCTTCA-3′, MUT seed sequence: 5′-AGTGAAGACTGAAGAACCTG-3′). To generate the mutant *MMP1* mRNA sequence, we mutated seven nucleotides of the seed region (WT seed sequence: 5′-AGCTTCA-3′; MUT seed sequences:5′-GATCCTG-3′). RA-FLS (1×10^5) were transfected with either the WT or MUT *Circ_0088200*, miR-127-5p mimics or mimics control, and WT or MUT *MMP1* mRNA, using Lipofectamine 2000 (Thermo Fisher Scientific). After induction for 48 hours, luciferase activity was assessed using a dual-luciferase reporter kit (Promega).

Experimental CIA mouse model

Twenty male DBA/1 mice, aged 7-8 weeks, weighing 20 ± 2 g, were provided by Beijing Qingle Biological Co., Ltd. (Beijing, China). All animal-related experiments in this study were performed with approval from the Medical Ethics Committee of Nanfang Hospital and carried out in strict compliance with the animal ethics requirements. A model of CIA was induced by intradermal immunization with emulsions prepared using Bovine type II collagen (CII) and complete Freund's adjuvant (CFA, Sigma, Kawasaki, Japan) in a 1:1 ratio. Sixteen mice were subjected to intra-articular injection of the emulsions to induce RA on day 0 day and on day 21, and the remaining four mice were injected with PBS to serve as internal controls. The arthritis index (AI) was scored 2–3 times per week, and AI score ≥ 6 indicated

that the CIA model was constructed successfully. In the present study, CIA was induced successfully in 12 mice according to the AI scoring.

Articular injection of *Circ_0088200*

The Adeno-associated virus vector encoding *Circ_0088200* (AAV-*Circ_0088200*) and Adeno-associated virus empty vector were constructed and packaged by Hanbio Biotechnology. After determining the AI and selecting the successful CIA models, the 12 CIA mice were randomly divided into three groups (injection of the normal control, injection of AAV-*Circ_0088200*, and injection of the AAV empty vector), with four mice in each group. A 10 μl solution containing the experimental virus (overexpressing human *Circ_0088200*) or control virus (both at approximately1.3 $\times 10^{12}$ viral genomes (vg)/ml) was slowly injected into each knee joint on the 28 day. The normal control group was injected with same volume of PBS. The injection procedure was repeated after 1 week and the mice were sacrificed at 60 days. The bilateral knees were dissected out and used for histological analysis and micro computed tomography (microCT) analysis.

Supplementary Table 1. Clinical characters and laboratory measures of the participants.

	**RA (*n* = 9)**	**OA (*n* = 7)**
Age (year, mean±SD)	51.11±2.72	51.71±2.10
Gender (male/female)	2/7	2/5
Anti-CCP* (U/ml)	37.59 (7.80-1121.50)	NA*
DAS28* (scores)	7.01 (3.08-7.71)	NA*

*DAS28: disease activity score in 28 joints; anti-CCP: anti-cyclic citrullinated peptide antibodies; NA: not available.

Supplementary Table 2. Primers and sequences used in this study.

Name	Sequence
Hsa_circ_0088200	F:5'TGCTGAACGAACTGCCCATA3'
	R:5'ATTTCAGGTTCTTTGGCTGTCG3'
TNC mRNA	F:5'CAACCATCACTGCCAAGTTCACAAC3'
	R:5'CGCCTCAGCCTTATCACCATTCAG3'
MMP1 mRNA	F:5′GGGCTGAAAGTGACTGGGAAAC3′
	R:5′CTTGGCAAATCTGGCGTGTA3′
GAPDH	F:5'GGGAAACTGTGGCGTGAT3'
	R:5'GAGTGGGTGTCGCTGTTGA3'
Si-hsa_circ_0088200_001	CACAGCCACGACAGCCAAA
Si-hsa_circ_0088200_002	AGCCACGACAGCCAAAGAA
Si-hsa_circ_0088200_003	CACGACAGCCAAAGAACCT
Si-MMP1-1	GCTTGAAGCTGCTTACGAA
Si-MMP1-2	GGACCATGCCATTGAGAAA
Si-MMP1-3	GCACATGACTTTCCTGGAA
MiR-127-5p	F:5'CTGAAGCTCAGAGGGCTCTGAT3'
	R: Universal Reverse Primer
U6	F:5'GGAACGATACAGAGAAGATTAGC3'
	R:5'TGGAACGCTTCACGAATTTGCG3'
MiR-127-5p mimics	F:5'CUGAAGCUCAGAGGGCUCUGAU3'
	R:5'AUCAGAGCCCUCUGAGCUUCAG3'
MiR-127-5p inhibitors	AUCAGAGCCCUCUGAGCUUCAG
